# MRE11 UFMylation promotes ATM activation

**DOI:** 10.1093/nar/gkz110

**Published:** 2019-02-20

**Authors:** Zhifeng Wang, Yamin Gong, Bin Peng, Ruifeng Shi, Dan Fan, Hongchang Zhao, Min Zhu, Haoxing Zhang, Zhenkun Lou, Jianwei Zhou, Wei-Guo Zhu, Yu-Sheng Cong, Xingzhi Xu

**Affiliations:** 1 Guangdong Key Laboratory for Genome Stability & Disease Prevention, Shenzhen University School of Medicine, Shenzhen, Guangdong 518060, China; 2 Department of Molecular Cell Biology and Toxicology, School of Public Health, Nanjing Medical University, Nanjing, Jiangsu 211166, China; 3 Shenzhen University-Friedrich Schiller Universität Jena Joint PhD Program in Biomedical Sciences, Shenzhen University School of Medicine, Shenzhen, Guangdong 518060, China; 4 College of Life Sciences, Capital Normal University, Beijing 100080, China; 5 College of Life Sciences & Oceanography, Shenzhen University, Shenzhen, Guangdong 518060, China; 6 Department of Oncology, Mayo Clinic, Rochester MN 55905, USA; 7 Institute of Aging Research, School of Medicine, Hangzhou Normal University, Hangzhou, Zhejiang 310036, China

## Abstract

A proper DNA damage response (DDR) is essential to maintain genome integrity and prevent tumorigenesis. DNA double-strand breaks (DSBs) are the most toxic DNA lesion and their repair is orchestrated by the ATM kinase. ATM is activated via the MRE11–RAD50–NBS1 (MRN) complex along with its autophosphorylation at S1981 and acetylation at K3106. Activated ATM rapidly phosphorylates a vast number of substrates in local chromatin, providing a scaffold for the assembly of higher-order complexes that can repair damaged DNA. While reversible ubiquitination has an important role in the DSB response, modification of the newly identified ubiquitin-like protein ubiquitin-fold modifier 1 and the function of UFMylation in the DDR is largely unknown. Here, we found that MRE11 is UFMylated on K282 and this UFMylation is required for the MRN complex formation under unperturbed conditions and DSB-induced optimal ATM activation, homologous recombination-mediated repair and genome integrity. A pathogenic mutation MRE11(G285C) identified in uterine endometrioid carcinoma exhibited a similar cellular phenotype as the UFMylation-defective mutant MRE11(K282R). Taken together, MRE11 UFMylation promotes ATM activation, DSB repair and genome stability, and potentially serves as a therapeutic target.

## INTRODUCTION

DNA damage introduced by endogenous and exogenous factors poses a serious hazard to cell viability and genome stability. A proper DNA damage response (DDR) is essential to maintain genome integrity and prevent tumorigenesis. In eukaryotic cells, maintenance of genomic stability relies on the coordinated action of a network of cellular processes collectively known as DDR. DNA double-strand breaks (DSBs) are the most toxic DNA lesion and their repair is orchestrated by ATM kinase. ATM is activated by DNA ends in the presence of the MRE11–RAD50–NBS1 (MRN) complex (1–3), along with its autophosphorylation at S1981 (4) and acetylation at K3106 (5). Activated ATM rapidly phosphorylates a vast number of substrates in local chromatin, providing a scaffold for the assembly of higher-order complexes that can repair damaged DNA (6).

Ubiquitination is one of the most common post-translational modifications, next to glycosylation and phosphorylation (7). A family of ubiquitin-like proteins (UBLs) has been identified that shows structural similarities to ubiquitin. It is generally believed that protein modification by UBLs serves mainly proteolysis-independent events, such as molecular assembly and the functional conversion of proteins (8). Ubiquitin-fold modifier 1 (UFM1) is the newest addition to the UBLs (9). Similar to ubiquitination, UFM1 conjugates to target protein(s) via the E1 and E2-like enzymes UBA5 and UFC1 and the E3 ligase UFL1 (10). Although two specific UFM1-specific proteases have also been identified, UfSP1 and UfSP2, humans only express one functional enzyme UfSP2 (11). The UFMylation pathway has been demonstrated to modulate several cellular activities, including endoplasmic reticulum stress, hematopoiesis, fatty acid metabolism, G-protein-coupled receptor biogenesis and neurodevelopment, with a limited number of physiological substrates identified (10,12). While reversible ubiquitination plays an important role in the DSB response (12–14), post-translational modification by UFM1 and its potential function in the DDR is largely unknown (12).

Here, we reported that MRE11 is UFMylated on K282 and this UFMylation is required for optimal ATM activation, homologous recombination-mediated DSB repair and genome integrity. A pathogenic mutation MRE11(G285C) identified in uterine endometrioid carcinoma exhibited a similar cellular phenotype as the UFMylation-defective mutant MRE11(K282R). Our findings demonstrated that the UFMylation pathway and MRE11 UFMylation may potentially serve as a therapeutic target.

## MATERIALS AND METHODS

### Cell culture, plasmid construction, drugs and ionizing raidiation

Human U2OS, DU145, A549 and HEK293T cells were cultured at 37°C with 5% CO_2_ in Dulbecco’s modified Eagle’s medium supplemented with 10% fetal bovine serum and 1% penicillin/streptomycin.

MRE11, UFL1 and UfSP2 complementary DNA (cDNAs) were sub-cloned into lenti-blast-vectors (Novobio). MRE11 cDNA was cloned into the EGFP-C1 expression vector (Clontech) and NBS1 cDNA was cloned into the EGFP-N1 expression vector (Clontech). UFM1 cDNA with two amino acids deleted in the C-terminal (UFM1ΔC2) and UfSP2 cDNA were cloned into the pcDNA3.0-HA vector. UBA5, UFC1, UFL1 and UfBP1 cDNAs were cloned into the vector with a MYC epitope. Point mutations in MRE11 and UfSP2(C302S), and small hairpin RNA (shRNA) target site-resistant mutations in UFL1 were generated using the Mut Express II Fast Mutagenesis Kit V2 (Vazyme). Bacteria expressing HIS-tagged UBA5, UFC1, UFL1, UFM1ΔC2 and GST-tagged MRE11 were generated using the pET28a (Invitrogen) and pGEX-4T-1 (GE Healthcare) systems, respectively.

Nocodazole (M1404) was purchased from Sigma; puromycin (S7417) was purchased from Selleck; bleomycin (H20055883) was purchased from Hisun Pfeizer; blasticidin S HCl (R210-01) was purchased from Invitrogen.

Cells were irradiated with a Rad Source RS-2000pro X-Ray irradiator at a dose rate of 1.67 gray (Gy)/min.

### RNAi

Endogenous UFL1 expression was knocked down using the following siRNAs: siUFL1 1#, 5′-GUUCCAACAUCGACAAGCA-3′; siUFL1 2#, 5′-CAGGGAGAUUAUCCCUUGA-3′. The siRNAs were transfected into cells by lipofectamine RNAiMAX (Invitrogen).

### Stable cell line establishment

Endogenous UFL1 (shUFL1) or MRE11 (shMRE11) silencing in A549, DR-U2OS or EJ5-GFP U2OS cells was achieved by infection of shRNA lentiviral constructs within the pLKO.1 vector and subsequent selection in puromycin. The shRNA sequences were as follows: shUFL1, 5′-GUUCCAACAUCGACAAGCA-3′; shMRE11, 5′-GAGCAUAACUCCAUAAGUA-3′ (15). The stably silenced cells were reconstituted with an shRNA-resistant form of FLAG-UFL1, FLAG-MRE11, FLAG-MRE11(K282R) or FLAG-MRE11(G285C) through lentiviral infection and blasticidin selection. Stable expression of FLAG-UfSP2 and FLAG-UfSP2(C302S) in cells was also achieved by lentiviral infection and subsequent blasticidin selection.

### Immunoblotting, immunoprecipitation and immunostaining

Anti-HA (A190-208A), anti-MYC (A190-205A), anti-UFL1 (A303-455A, A303-456A), anti-RAD50 (A300-184A), anti-NBS1 (A300-187A) and anti-MRE11 (A300-181A) were purchased from Bethyl. Anti-FLAG (F1804), anti-Tubulin (T5168) and anti-Actin (A5441) were purchased from Sigma. Anti-GFP (sc-9996) was purchased from Santa Cruz. Anti-HIS (D291-3), anti-GFP (D153-3) and anti-GST (M209-3) were purchased from MBL. Anti-γH2AX (05-636) was purchased from Millipore.

Immunoblotting and immunoprecipitation (IP) were performed as previously described (16). IP in denatured conditions (Sodium dodecyl sulphate (SDS)-IP) was also performed as previously described (11). Briefly, the harvested cells were lysed in lytic buffer (150 μM Tris, pH 8.0, 5% SDS, 30% glycerol) at 100°C for 5 min before digestion with benzonuclease (Sigma) at room temperature for 30 min. The lysates were centrifuged at high speed and then diluted 20× with buffer 150 (50 mM Tris, pH 8.0, 5 mM ethylenediaminetetraacetic acid, 150 mM NaCl, 0.5% nonidet P-40 (NP-40), protease inhibitor cocktail) and immunoprecipitated with the appropriate antibodies.

Co-immunostaining of γH2AX and UFL1 was performed as previously described (17). Briefly, U2OS cells were micro-irradiated with a MicroPoint System (Andor) and sequentially fixed with 4% paraformaldehyde (PFA) at room temperature for 10 min followed by cold methanol at 4°C for 20 min. A γH2AX mouse monoclonal antibody and rabbit UFL1 polyclonal antibody was used for co-immunostaining. Images were captured using a DragonFly confocal imaging system (Andor).

### Chromatin fractionation

Chromatin fractionation was performed as previously described (18).

### Protein purification

Protein expression and purification were performed as previously described (19). GST-fused MRE11 and MRE11(K282R) were ectopically expressed in BL21 cells and purified using glutathione-Sepharose 4B (GE Healthcare). HIS-UBA5, HIS-UFC1, HIS-UFL1 and HIS-HA-UFM1ΔC2 were expressed in BL21 cells and purified using Ni-NTA agarose (Qiagen).

### *In vitro* UFMylation assay

*In vitro* UFMylation assay was performed as previously described (20). Briefly, purified HIS-UBA5 (0.1 μM), HIS-UFC1 (0.1 μM), HIS-UFL1 (0.1 μM), HIS-HA-UFM1ΔC2 (0.1 μM) and GST-MRE11 (0.1 μM) were mixed in a reaction buffer (0.05% bovine serum albumin, 50 mM 4-(2-hydroxyethyl)-1-piperazineethanesulfonic acid (HEPES), pH 7.5) containing 5 mM γ-adenosine triphosphate (ATP) and 10 mM MgCl_2_ and incubated at 30°C for 90 min. The mixtures were then boiled in SDS sample buffer containing 5% 2-mercaptoethanol for 10 min.

### DSBs induced by micro-irradiation with a UV laser beam

U2OS or DU145 cells expressing the indicated GFP-tagged proteins were seeded in glass-bottomed confocal dishes (Nest). DSBs were generated using a Micropoint System (Andor) by 365 nm pulsed nitrogen Ultraviolet (UV) laser (16 Hz pulse, 60% output). Images were captured in real time every 10 s under a DragonFly confocal imaging system (Andor). The fluorescence intensity was determined with ImageJ (NIH). The data represent the means of the at least 10 independent measurements.

### HR-mediated DSB repair

HR-mediated repair of DSBs using DRU2OS cells in which a single copy of DRGFP reporter gene has been integrated into its genome, was performed as previously described (21,22).

### Mitotic spread analysis

Mitotic spread assays were performed as previously described (23,24). Briefly, A549 cells were depleted of UFL1 or MRE11 and then reconstituted with FLAG-UFL1 or FLAG-MRE11, FLAG-MRE11(K282R) or FLAG-MRE11(G285C). The cells were then treated with 10 μM nocodazole for 6 h before harvesting. Chromosome spreads were prepared after treating the cells with a hypotonic solution containing 56 mM potasium chloride (KCl), and then fixed in methanol/acetic acid (volume ratio of 3:1) and stained with Giemsa. The images were captured under a DragonFly confocal imaging system (Andor). For each experiment, >400 mitotic chromosomes were randomly selected and analyzed.

### Statistical analysis

Quantification data were analyzed by performing a two-way analysis of variance (ANOVA) or a Spearman’s rank correlation test. A *P* < 0.05 were considered statistically significant.

## RESULTS

### UFL1/UfSP2 dynamically interacts with the MRN complex in response to DNA damage

**Figure 1.**
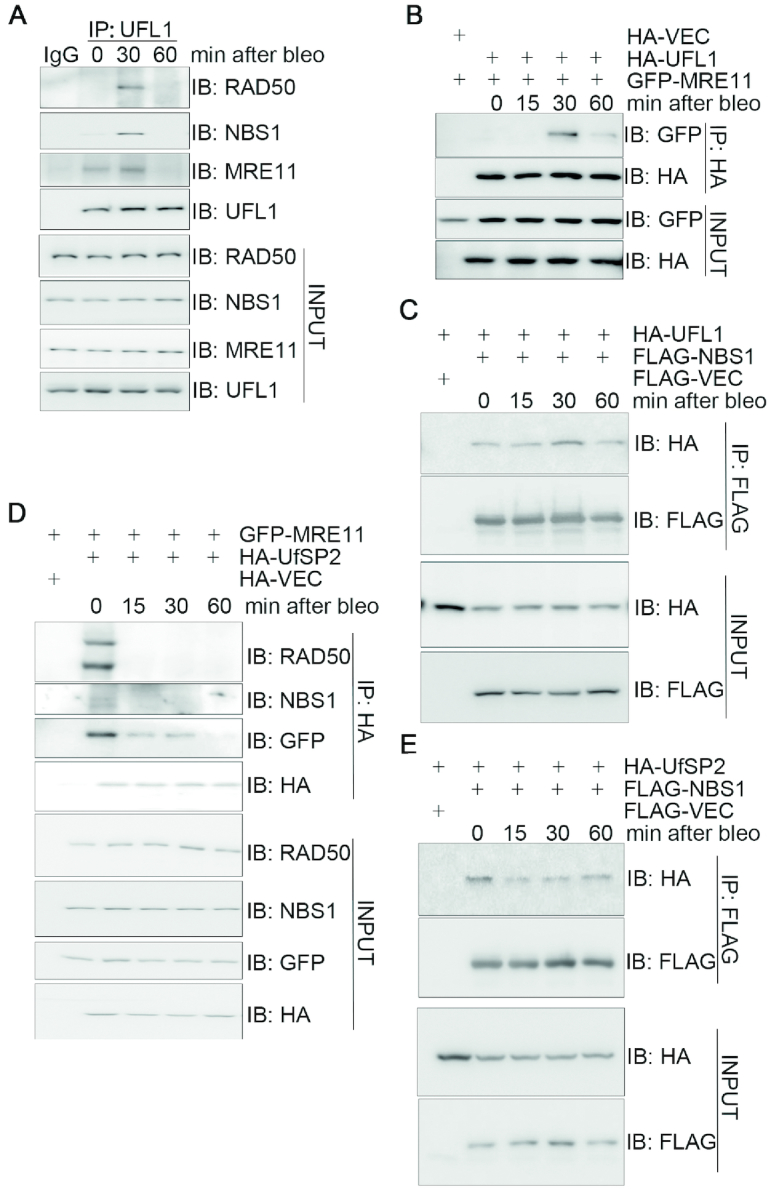
UFL1/UfSP2 dynamically interacts with the MRN complex in response to DNA damage. (**A**) DSB-induced interaction between UFL1 and the MRN complex. HEK293T cells were treated with 10 μg/ml bleomycin and harvested at the indicated time points. Total cell lysates were subjected to IP followed by immunoblotting with the indicated antibodies. (**B**) The interaction between UFL1 and MRE11 peaked 30 min after bleomycin treatment. HEK293T cells co-expressing HA-UFL1 and GFP-MRE11 were treated with 10 μg/ml bleomycin at the indicated time points. Total cell lysates were harvested and subjected to IP and immunoblotting with the indicated antibodies. (**C**) The interaction between HA-UFL1 and FLAG-NBS1 peaked at 30 min after bleomycin treatment. HEK293T cells co-expressing HA-UFL1 and FLAG-NBS1 were treated with 10 μg/ml bleomycin. Total cell lysates were harvested at the indicated times and subjected to IP and immunoblotting with the indicated antibodies. (**D** and **E**) DNA damage disrupted the interaction between HA-UfSP2 and GFP-MRE11 or FLAG-NBS1. HEK293T cells co-expressing HA-UfSP2 and GFP-MRE11 or FLAG-NBS1 were treated with 10 μg/ml bleomycin. Total cell lysates were harvested at different times as indicated, and subjected to IP and immunoblotting with the indicated antibodies. Abbreviations: bleo, bleomycin; IB, immunoblot; IP, immunoprecipitation.

To explore whether the UFMylation pathway has a role in the DDR, we examined whether UFMylation factors (UFM1, UBA5, UFC1, UFL1 and UfSP2) could be enriched at UV laser (365 nm)-induced DNA damage stripes in U2OS cells. Immunofluorescence staining showed that endogenous UFL1 co-localized with the DSB marker γH2AX on the DNA damage stripe (Supplementary Figure S1A). In addition, chromatin fractionation assays uncovered that both bleomycin treatment and X-ray irradiation induced an increase of UFL1 in the chromatin-enriched fraction in U2OS cells (Supplementary Figure S1B). These results prompted us to perform a candidate screen for potential interactions between UFL1 and DDR factors. Here, we found that endogenous MRE11, RAD50 and NBS1 were present in the endogenous UFL1 immunocomplex (Figure 1A) and that these interactions peaked at 30 min and returned to the basal levels 60 min after bleomycin treatment (Figure 1A). A similar interaction pattern was observed for the epitope-tagged MRE11 or NBS1 with HA-UFL1 (Figure 1B and C). We also found that X-ray irradiation (5 Gy) induced the interaction between GFP-MRE11 and HA-UFL1 and this interaction peaked at 15 min and returned to basal levels 40 min after irradiation (Supplementary Figure S2A). Further co-IP assays revealed that GFP-MRE11 and FLAG-NBS1 were present in the immunocomplex of HA-UfSP2 in HEK293T cells and these co-IPs diminished upon bleomycin treatment (Figure 1D and E) or 5-Gy ionizing radiation (IR) (Supplementary Figure S2B and data not shown). These results revealed that UFL1/UfSP2 physically interact with the MRN complex and that these interactions are dynamically modulated by DNA damage, implying a functional interplay between UFMylation pathway and the MRN complex.

### UFMylation promotes MRN complex formation under unperturbed conditions and MRE11/NBS1 recruitment to the DNA damage stripes

**Figure 2.**
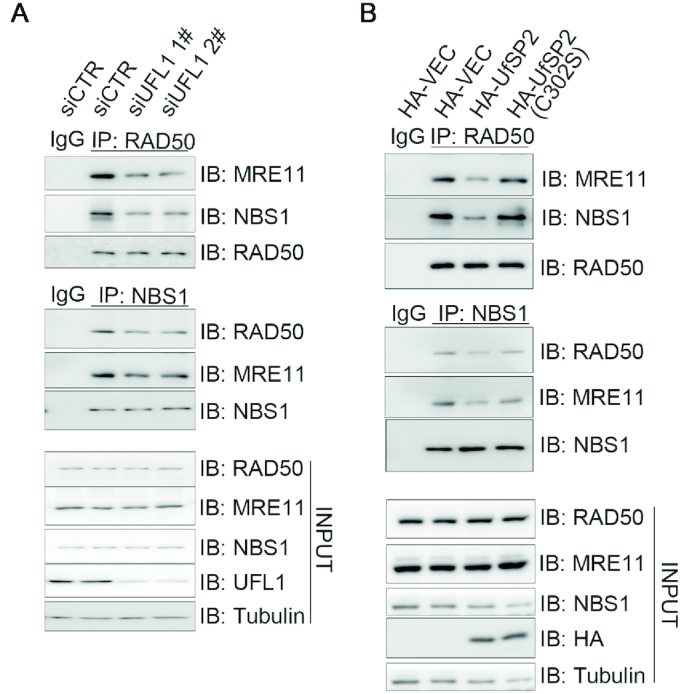
UFMylation promotes MRN complex formation under unperturbed conditions. (**A**) UFL1 depletion decreased MRN complex formation. Total lysates derived from HEK293T cells transfected with UFL1 (siUFL1 1# and 2#) or control (siCTR) siRNAs were immunoprecipitated with RAD50 or NBS1 antibodies. The precipitates were probed with the indicated antibodies. (**B**) Over-expression of UfSP2 compromised MRN complex formation. Total lysates derived from HEK293T cells overexpressing HA-VEC, HA-UfSP2 or HA-UfSP2(C302S) were subjected to IP and immunoblotting with the indicated antibodies. Abbreviations: bleo, bleomycin; IB, immunoblot; IP, immunoprecipitation; siCTR, small interfering RNA control.

We next explored whether UFMylation impacts on MRN function. Indeed, inhibition of UFL1 expression with two independent siRNAs led to a reduction of the endogenous level of MRE11 or NBS1 present in the RAD50 immunocomplex under unperturbed conditions (Figure 2A). Similarly, expression of HA-UfSP2, but not the catalytically inactive mutant HA-UfSP2(C302S), reduced the interaction between MRE11 or NBS1 and RAD50 (Figure 2B). These data indicated that UFMylation is required for MRN complex formation under physiological conditions.

**Figure 3.**
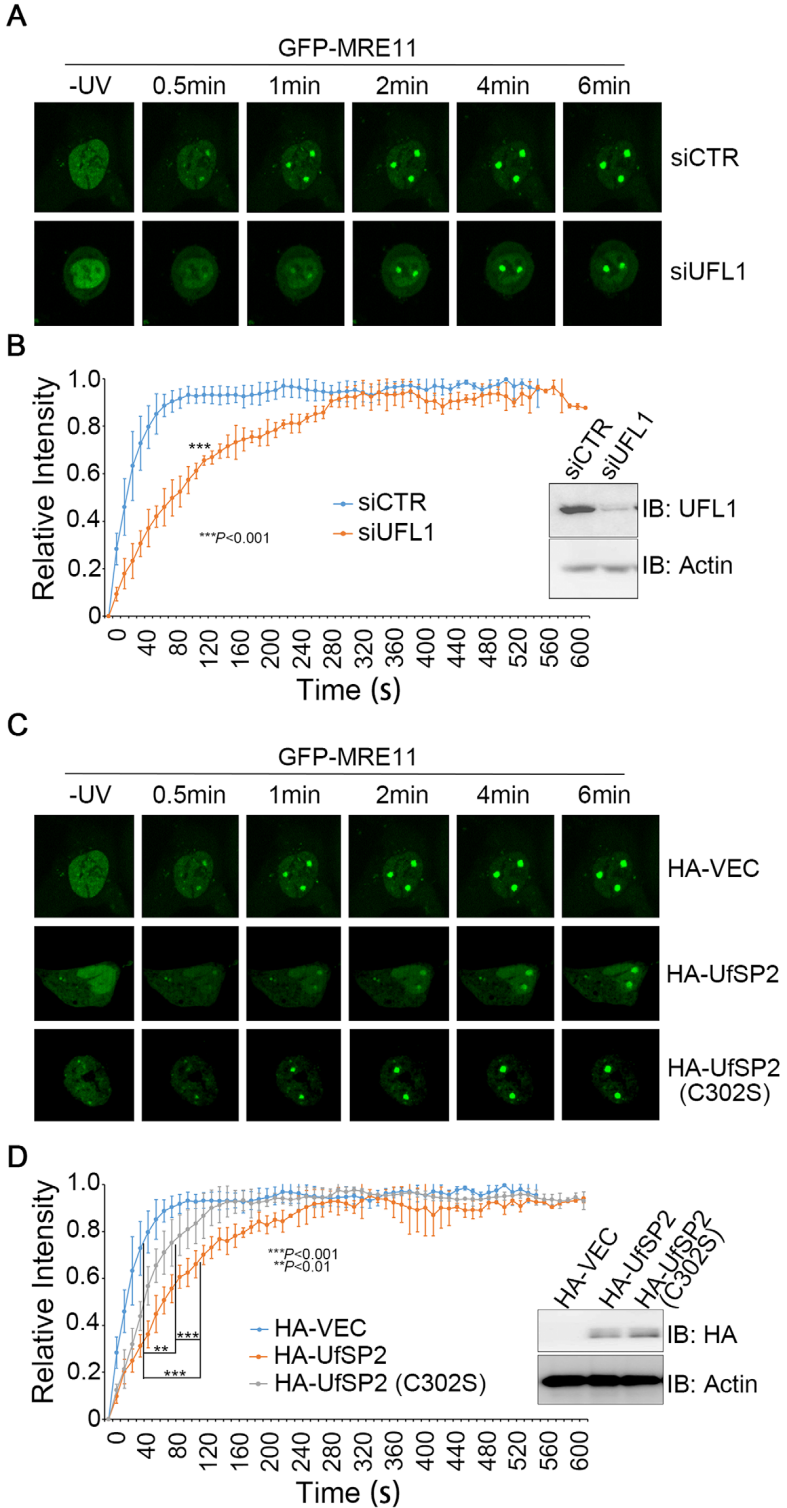
UFMylation promotes MRE11 recruitment to the DNA damage stripes. (**A** and **B**) UFL1 depletion decreased the initial recruitment of MRE11 to DNA damage stripes. UFL1-depleted (siUFL1) or mock (siCTR)-depleted DU145 cells transiently expressed GFP-MRE11. (**C** and **D**) UfSP2 over-expression decreased MRE11 initial recruitment to DNA damage stripes. DU145 cells were co-transfected with an HA vector, HA-UfSP2 or HA-UfSP2(C302S) and GFP-MRE11 at a molar ratio of 10:1. GFP-positive cells were micro-irradiated with a UV laser (365 nm) and consecutive images were collected at 10-s interval for 10 min. Representative images of GFP-MRE11 recruitment are shown in A and C, and the statistical analysis of recruitment dynamics with the Spearman’s rank-order correlation test is shown in B and D.

Given that the MRN complex binds to DSB and initiates the DSB end processing prior to repair (25), we examined the impact of UFMylation on the recruitment of the complex to the DNA damage sites. To this end, we transiently expressed GFP-MRE11 or NBS1-GFP in U2OS cells depleted of UFL1 by siRNA. These transfectants were irradiated by a UV laser beam (wavelength of 365 nm) and the recruitment kinetics of GFP-MRE11 and NBS1-GFP were monitored live every 10 s up to 10 min. It was found that inhibition of UFL1 expression compromised the initial recruitment, but not retention, of GFP-MRE11 (Figure 3A and B) and NBS1-GFP (Supplementary Figure S3A and B) onto the UV laser irradiation-induced DNA damage stripes. A similar effect was observed upon HA-UfSP2, but not HA-UfSP2(C302S) expression, on GFP-MRE11 (Figure 3C and D) and NBS1-GFP (Supplementary Figure S3C and D) recruitment. These findings demonstrated that UFMylation ensures timely recruitment of the MRN complex to the DNA damage sites.

We then sought to compare the recruitment kinetics of UFL1 to those of MRE11. Although we were able to observe endogenous UFL1 enrichment on the UV laser-induced DNA damage stripes (Supplementary Figure S1A), we failed to detect GFP-UFL1 or UFL1-GFP recruitment to the DNA damage stripes (data not shown). We thus turned to examine focus formation of endogenous UFL1. We found that 5-Gy IR induced UFL1 focus formation, and the number of UFL1 foci that co-localized with γH2AX per cell peaked at 10 min after IR (Supplementary Figure S4A), while the number of MRE11 foci that co-localized with γH2AX per cell steadily increased up to 1 h after IR (Supplementary Figure S4B and C). Although DNA damage-induced initial recruitment of MRE11 to the DNA damage stripes is UFL1-depednent (Figure 3 and Supplementary Figure S3), it is still not conclusive if UFL1 is recruited to the DNA damage site earlier than the MRN complex. Nevertheless, our data indicate that UFL1 is enriched transiently at the damage site at the early stage of DDR.

We further explored whether MRE11 confers a feedback regulation of UFL1 recruitment to the DNA damage site. To this end, A549 cells with mock knockdown or stable MRE11 knockdown with or without reconstitution of wild-type FLAG-MRE11 were irradiated by 5-Gy IR. It was found that inhibition of MRE11 expression by shRNA reduced the number of UFL1 foci per cell that co-localized with γH2AX to about 1/3 and this reduction was almost fully restored upon re-expressing an shRNA-resistant FLAG-MRE11 (Supplementary Figure S5). Together, these data demonstrated that DNA damage-induced recruitment of UFL1 and the MRN complex to the damage site is inter-dependent.

### Defective UFMylation compromises ATM activation upon DNA damage

**Figure 4.**
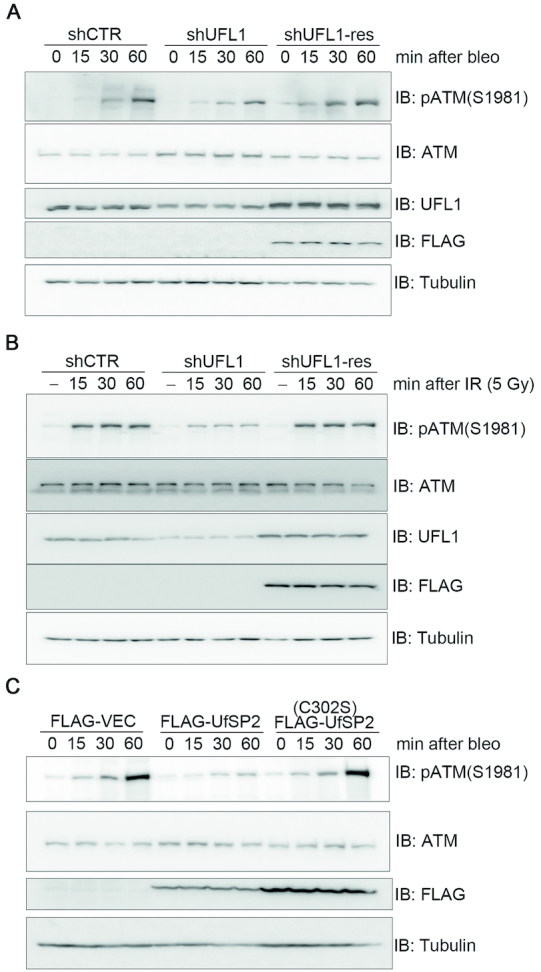
Defective UFMylation compromises optimal ATM activation upon DNA damage. (**A** and **B**) Inhibition of UFL1 expression compromised optimal activation of ATM upon DNA damage. A549 cells that were mock depleted (shCTR), UFL1 depleted (shUFL1) or UFL1 depleted, and then rescued with an shUFL1-resistant form of UFL1 (shUFL1-res) were treated with 10 μg/ml bleomycin (A) or irradiated with 5-Gy IR (B). Total cell lysates were harvested at different times after treatment and subjected to immunoblotting with the indicated antibodies. (**C**) Over-expression of UfSP2 compromised optimal activation of ATM upon DNA damage. A549 cells expressing FLAG-VEC, FLAG-UfSP2 or FLAG-UfSP2(C302S) were treated with 10 μg/ml bleomycin. Total cell lysates were harvested and subjected to immunoblotting with the indicated antibodies.

The MRN complex directly activates the ATM kinase in response to DSBs (1–3). We therefore reasoned that UFMylation could be important for DNA damage-induced ATM activation. To this end, we used a gene-specific shRNA to inhibit UFL1 expression and found that UFL1 depletion reduced ATM S1981 phosphorylation levels at early time points following bleomycin treatment or 5-Gy IR. This reduction was restored upon re-expression of an shRNA-resistant UFL1 (Figure 4A and B). Conversely, expression of FLAG-UfSP2, but not FLAG-UfSP2(C302S), reduced bleomycin-induced ATM phosphorylation levels at all time points (Figure 4C). Taken together, these results demonstrate that UFMylation ensures optimal activation of the ATM kinase in response to DSBs, probably through promoting the timely recruitment of the MRN complex to the DNA damage sites.

### MRE11 is UFMylated and this UFMylation transiently increases after DSB induction

**Figure 5.**
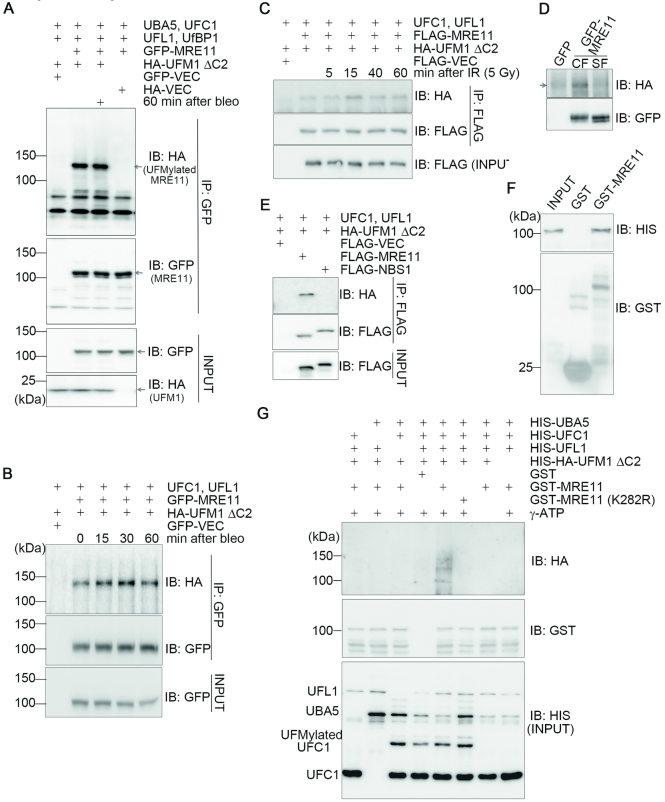
MRE11 is UFMylated and this UFMylation transiently increases after DSB induction. (**A**) MRE11 is UFMylated *in vivo*. HEK293T cells co-expressing UFMylation factors (UBA5, UFC1, UFL1, UfBP1, HA-UFM1ΔC2) and GFP-MRE11 were lysed in 5% SDS-containing buffer. Total cell lysates were boiled for 5 min before IP and immunoblotting with the indicated antibodies. (**B** and **C**) MRE11 UFMylation is induced by DSBs. Experiments were performed as described in A except that HEK293T transfectants were treated with 10 μg/ml bleomycin (B) or 5-Gy IR (C) and total cell lysates were harvested at different time-points post-treatment. (**D**) UFMylated MRE11 is enriched on chromatin. Chromatin fractionation assays were performed with HEK293T cells co-expressing the UFMylation factors (UBA5, UFC1, UFL1, UfBP1, HA-UFM1ΔC2) and GFP-MRE11. Both the cytosolic fraction (SF) and chromatin-enriched fraction (CF) were subjected to IP with an anti-GFP antibody and immunoblotting with the indicated antibodies. (**E**) UFMylation of NBS1 was undetected. HEK293T cells co-expressing the UFMylation factors (UBA5, UFC1, UFL1, UfBP1, HA-UFM1ΔC2) and FLAG-MRE11 or FLAG-NBS1 were lysed in 5% SDS-containing buffer. Total cell lysates were boiled for 5 min before IP and immunoblotting with the indicated antibodies. (**F**) MRE11 directly interacted with UFL1 *in vitro*. Bacterially produced GST or GST-MRE11 was used to pull down bacterially produced HIS-UFL1. (**G**) MRE11 is UFMylated *in vitro*. Recombinant UFMylation factors (HIS-UBA5, HIS-UFC1, HIS-UFL1 and HIS-HA-UFM1ΔC2) with bacterially produced GST, GST-MRE11 or GST-MRE11(K282R) were incubated in UFMylation buffer at 30°C for 90 min. The reaction was terminated by adding SDS sample buffer and the samples were subjected to sodium dodecyl sulphate-polyacrylamide gel electrophoresis (SDS-PAGE) followed by immunoblotting with the indicated antibodies.

Because UFL1 physically interacts with the MRN complex and UFMylation promotes enrichment at DSBs (Figures 1–4), we speculated that the MRN complex or its subunit(s) could be targeted for UFM1 modification. To ensure breakdown of non-covalent conjugations to the target protein, we first extracted total cell lysates in 5% SDS-containing lysis buffer from HEK293T cells expressing the UFMylation enzymes along with GFP-MRE11 and HA-UFM1. The samples were boiled for 5 min before immunoprecipitating with an anti-GFP antibody. Immunoblotting with an anti-HA antibody revealed a slower migration form of MRE11, ∼20 kDa heavier than the predicted molecular weight of GFP-MRE11 (Figure 5A), suggestive of a covalent UFM1 modification. It was noted that MRE11 UFMylation peaked at 30 min and returned to basal levels 60 min after bleomycin treatment (Figure 5A and B). It was also noted that MRE11 UFMylation peaked at 15 min and returned to basal levels 40 min after 5-Gy IR (Figure 5C). These findings are consistent with the interaction kinetics between UFL1 and the MRN complex (Figure 1 and Supplementary Figure S2). Furthermore, hydrogen peroxide treatment at a final concentration of 4 mM in HEK293T cells for 10, 30 or 60 min did not lead to any obvious increase in MRE11 UFMylation (data not shown). Chromatin fractionation assays revealed that MRE11 UFMylation mainly occurred in the chromatin-enriched fraction (Figure 5D). Under the same conditions, we found that FLAG-MRE11, but not NBS1-FLAG, was UFMylated (Figure 5E). We next studied whether MRE11 could be UFMylated *in vitro*. We found that bacterially produced GST-MRE11 could pull down bacterially produced HIS-UFL1 (Figure 5F), indicating a direct interaction between MRE11 and UFL1. *Invitro*, UFMylation assays demonstrated that GST-MRE11 was UFMylated (Figure 5G). In sum, these results demonstrate that MRE11 is UFMylated both *in vivo* and *in vitro* and this UFMylation transiently increases mainly upon DSB induction.

### K282 is the essential UFMylation site in MRE11

**Figure 6.**
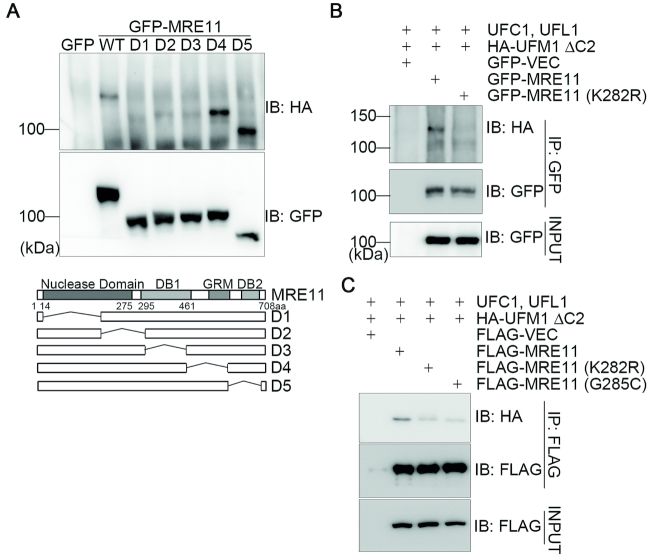
K282 is the essential UFMylation site in MRE11. (**A**) Mapping the essential regions for MRE11 UFMylation. A series of deletion mutants in the context of full-length MRE11 were generated as indicated. The mutants or the control plasmids were co-transfected with the UFMylation factors (UBA5, UFC1, UFL1, UfBP1, HA-UFM1ΔC2) into HEK293T cells. Experiments were performed as described in 5A but using these deletion mutants. (**B**) K282 is the major UFMylation site for MRE11. HEK293T cells co-expressing the UFMylation factors (UBA5, UFC1, UFL1, UfBP1, HA-UFM1ΔC2) and GFP-MRE11 or GFP-MRE11(K282R) were lysed in 5% SDS-containing buffer. Total cell lysates were boiled for 5 min before IP and immunoblotting with the indicated antibodies. (**C**) The pathogenic mutant MRE11(G285C) exhibited defective UFMylation. Experiments were performed as described in B except that GFP-MRE11(G285C) was also included.

To map the essential residue(s) for MRE11 UFMylation, we generated five deletion mutants in the context of full-length MRE11. We found that three deletion mutants spanning the first 430 amino acids of MRE11 resulted in an obvious reduction of UFMylation, indicating that potential UFMylation site(s) resided in the N terminus of MRE11 (Figure 6A). UFMylation assays of individual KR mutants revealed that the K282R mutation completely abolished MRE11 UFMylation *in vitro* (Figure 5G), while MRE11(K282R) UFMylation levels were diminished *in vivo* (Figure 6B). A Cancer Genome Atlas (TCGA) database search failed to identify any MRE11 mutations at K282; however, mutation of a surrounding residue (G285C) was found in uterine endometrioid carcinoma. *In vivo*, UFMylation assays uncovered that, while wild-type MRE11 was properly UFMylated, the UFMylation levels of MRE11(G285C) were markedly diminished to a similar level as the K282R mutant (Figure 6C). Taken together, these data reveal that MRE11 is UFMylated at K282 and a pathogenic mutation MRE11(G285C) compromises MRE11 UFMylation.

### Defective MRE11 UFMylation impairs MRN complex formation and DNA damage-induced ATM activation

**Figure 7.**
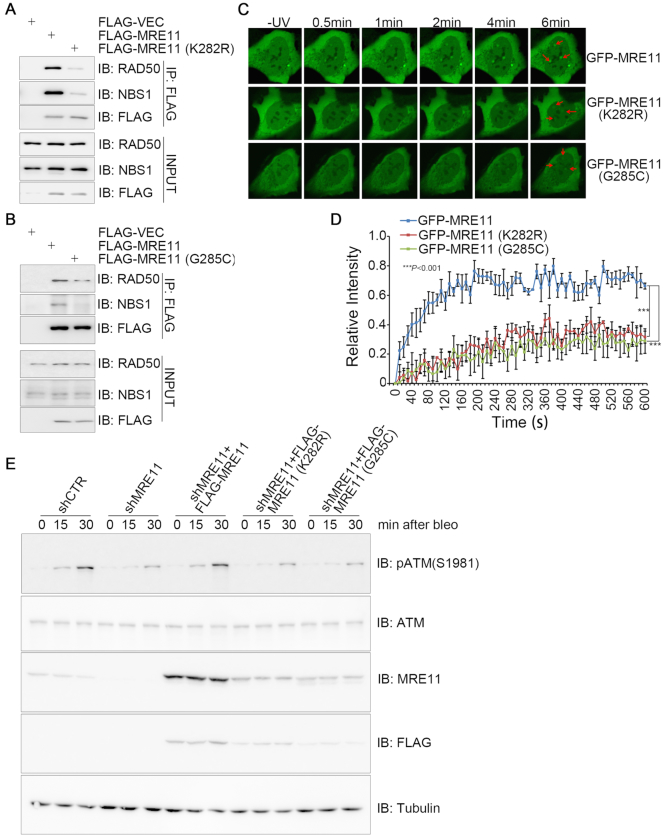
Defective MRE11 UFMylation impairs MRN complex formation and DNA damage-induced ATM activation. (**A** and **B**) UFMylation-defective mutants compromised formation of the MRN complex. Total cell lysates derived from HEK293T cells expressing a FLAG vector, FLAG-MRE11, FLAG-MRE11(K282R) or FLAG-MRE11(G285C) were subjected to IP with an anti-FLAG antibody and immunoblotting with the indicated antibodies. (**C** and **D**) UFMylation-defective mutants compromised recruitment of UFMylation-defective mutants to DNA damage stripes. U2OS cells transiently expressed GFP-MRE11, GFP-MRE11(K282R) or GFP-MRE11(G285C). GFP-positive cells were micro-irradiated with a UV laser (365 nm) and consecutive images were captured at 10-s interval for 10 min. Representative images of GFP-MRE11 recruitment are shown in C, and the statistical analysis of recruitment dynamics with the Spearman’s rank-order correlation test is shown in (D). (**E**) UFMylation-defective mutants compromised DNA damage-induced ATM activation. A549 cells stably expressing shCTR or shMRE11 were re-introduced with FLAG-MRE11, FLAG-MRE11(K282R) or FLAG-MRE11(G285C). The cells were treated with 10 μg/ml bleomycin for 15 or 30 min. Total cell lysates were harvested and analyzed by SDS-PAGE and immunoblotting with the indicated antibodies.

We then sought to determine the function of MRE11 UFMylation in physiological conditions and in the DDR. We found that lower levels of endogenous RAD50 and NBS1 were present in the FLAG-MRE11(K282R) or FLAG-MRE11(G285C) immunocomplex compared to the FLAG-MRE11 immunocomplex (Figure 7A and B), indicating that MRE11 UFMylation facilitates efficient MRN complex formation. Furthermore, we found that both GFP-MRE11(K282R) and GFP-MRE11(G285C) almost lost their capacity to localize to and maintain on UV laser irradiation-induced DNA damage stripes (Figure 7C and D). Lastly, inhibiting MRE11 expression in A549 cells slowed down ATM activation dynamics in response to bleomycin treatment (Figure 7E) or 5-Gy IR (Supplementary Figure S6); lentiviral-mediated re-expression of wild-type MRE11, but not the UFMylation-defective mutant MRE11(K282R) or MRE11(G285C), restored ATM activation (Figure 7E and Supplementary Figure S6). Taken together, these results reveal that MRE11 UFMylation promotes MRN complex formation under unperturbed conditions and ATM activation in response to DSB damage.

### MRE11 UFMylation promotes HR-medicated DSB repair and genome stability

**Figure 8.**
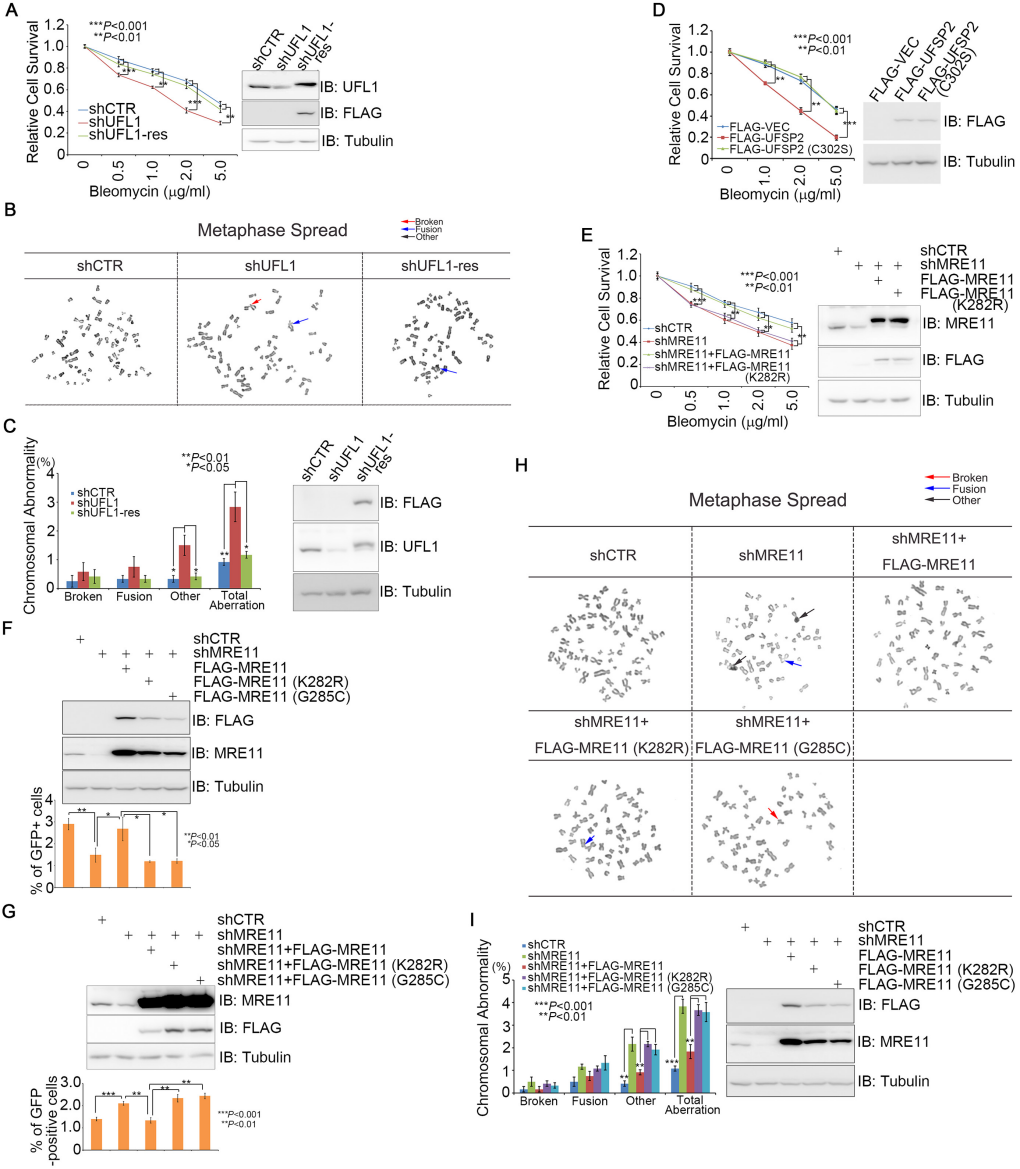
MRE11 UFMylation promotes HR-medicated DSB repair and genome stability. (**A**) Depletion of UFL1-sensitized cells to bleomycin treatment. A549 cells stably expressing shCTR, shUFL1 or shUFL1 reconstituted with an shUFL1-resistant form of FLAG-UFL1 were treated with increasing concentrations of bleomycin, as indicated. The surviving colonies were analyzed after 10 days. (**B** and **C**) Depletion of UFL1 resulted in aberrant mitotic chromosomes. The cells generated in A were used for mitotic spread preparation, and >400 mitotic chromosomes per type of manipulated cells were analyzed. Representative images of mitotic spreads are shown in B, while the quantitative analysis is shown in (C). (**D**) Overexpression of UfSP2 sensitized cells to bleomycin treatment. A549 cells stably expressing a FLAG vector, FLAG-UfSP2 or FLAG-UfSP2(C302S) were treated with increasing concentrations of bleomycin, as indicated. The surviving colonies were analyzed after 10 days. (**E**) MRE11(K282R) failed to rescue MRE11-depletion-induced cellular sensitivity to bleomycin treatment. A549 cells stably expressing shCTR, shMRE11, shMRE11 reconstituted with FLAG-MRE11 or FLAG-MRE11(K282R) were treated with increasing concentrations of bleomycin, as indicated. The surviving colonies were analyzed after 10 days. (**F**) Defective MRE11 UFMylation compromised HR-mediated DSB repair. DRU2OS cells, in which a DR-GFP reporter cassette is integrated, stably expressing shCTR, shMRE11 or shMRE11 reconstituted with FLAG-MRE11, FLAG-MRE11(K282R) or FLAG-MRE11(G285C). HR assays were performed in triplicate. (**G**) Defective MRE11 UFMylation promoted NHEJ-mediated DSB repair. U2OS cells, in which an EJ5-GFP reporter cassette is integrated, stably expressing shCTR, shMRE11 or shMRE11 reconstituted with FLAG-MRE11, FLAG-MRE11(K282R) or FLAG-MRE11(G285C). NHEJ assays were performed in triplicate. (**H** and **I**) Defective MRE11 UFMylation contributed to aberrant mitotic chromosomes. A549 cells stably expressing shCTR, shMRE11 or shMRE11 reconstituted with FLAG-MRE11, FLAG-MRE11(K282R) or FLAG-MRE11(G285C) and then subjected to mitotic spread preparation; >400 mitotic chromosomes per type of manipulated cells were analyzed. All data were derived from three independent experiments. Representative images of mitotic spreads are shown in H, while the quantitative analysis is shown in I. A two-way ANOVA was performed to determine statistical significance. *: p<0.05; **: p<0.01; ***: p<0.001.

Finally, we explored whether defective UFMylation results in genome instability. Clonogenic survival assays in A549 cells revealed that shRNA-mediated UFL1 inhibition sensitized cancer cells to bleomycin treatment (Figure 8A) and mitotic spread assays showed that UFL1 depletion resulted in an increase of aberrant mitotic chromosomes (Figure 8B and C), while re-expression of a shRNA-resistant form of UFL1 rescued this defect (Figure 8A–C). Reciprocally, stable expression of UfSP2, but not UfSP2(C302S), decreased cell viability after bleomycin treatment (Figure 8D). As expected, given that K282 is the essential site of MRE11 UFMylation, we found that MRE11 depletion sensitized cells to bleomycin treatment, while re-expression of wild-type MRE11 rescued this phenotype, whereas re-expression of the MRE11(K282R) UFMylation defective mutant resulted in a similar level of sensitivity to bleomycin treatment as MRE11-depleted cells (Figure 8E). Furthermore, inhibiting MRE11 expression resulted in defective HR-mediated DSB repair (Figure 8F), while moderately promoting non-homologous end joining (NHEJ)-mediated DSB repair probably due to defective HR (Figure 8G) and aberrant mitotic chromosomes (Figure 8H and I); re-expression of wild-type MRE11 rescued this defect, whereas re-expression of MRE11(K282R) or MRE11(G285C) failed to do so (Figure 8F–I). The biological significance of MRE11 we observed here is consistent with earlier reports (26,27). Taken together, these results demonstrate that MRE11 UFMylation promotes HR-mediated DSB repair and chromosome stability.

## DISCUSSION

UFMylation has been linked to several cellular processes (28–32). Amplification, deletion or mutation of genes encoding the UFMylation factors (UBA5, UFC1, UFL1, UfSP2 and UFM1) has been detected in malignant tumors from various tissues and organs in the TCGA database (10,12), indicating that UFMylation may promote or suppress tumorigenesis in different cellular contexts. Thus far, the detailed mechanisms underlying UFMylation behavior during genome integrity maintenance have been largely unknown. Here, we uncover that UFMylation promotes optimal activation of the ATM kinase, which subsequently contributes to the maintenance of chromosome stability.

ATM kinase is the master regulator of the DDR cascades and is thus subjected to delicate, multi-layered regulation (4,5,33). Once DNA damage occurs, ATM autophosphorylates to convert from an inactive dimer to an active monomer; it then phosphorylates a large number of functional substrates involved in cell-cycle checkpoints, DNA repair and various other cellular processes that ensure optimal efficacy of the signaling cascades (6,34,35). In the context of DSBs, the presence of both the MRN complex and processed DNA ends is essential to activate the ATM kinase (1–3,36). The MRN complex binds to DNA and forms stable complexes with the broken DNA ends through multiple interfaces, which unmask a normally inaccessible DNA-binding surface on ATM (37,38). Consequently, ATM is recruited to the DNA break sites where it is sufficiently active. In our study, we found that DSB-induced optimal ATM activation requires MRE11 UFMylation (Figures 4 and 7E). We showed that, following DNA damage, MRE11 is UFMylated at K282 both *in vitro* and *in vivo* (Figures 5G and 6B). Furthermore, UFMylated MRE11 is required for MRN complex assembly, and DNA damage-induced UFMylation of MRE11 facilitates recruitment of the MRN complex to the DNA damage site. This process helps relieve ATM kinase auto-inhibition at DSBs, thus promoting subsequent HR-mediated DSB repair and chromosome stability. In sum, our data provide a novel mechanism of ATM activation through MRE11 UFMylation.

The initial discovery that ATM was functionally connected to the MRN complex stemmed from the observation that patients with a rare clinical phenotype similar to that observed in patients with A-T-like disorder (ATLD) have mutations in *MRE11* (39). In addition, *MRE11* mutations were detected in various tumors, but the underlying pathologic mechanisms were unclear (38,40,41). Some components of the UFMylation cascade were also reported to be dysfunctional in several types of cancers (10,42–44), but the UFMylation substrates and the involved signaling pathway remain unidentified. Here, we showed that a pathogenic MRE11(G285C) mutation detected in uterine endometrioid carcinoma (TCGA database) exhibited a similar cellular phenotype with the UFMylation-defective mutant MRE11(K282R) (Figures 7 and 8), suggesting that the role of the MRE11 UFMylation is closely associated with tumorigenesis. In summary, our finding implies that targeting the UFMylation cascade may be a novel and promising therapeutic strategy for certain cancer types.

## Supplementary Material

Supplementary Data
