# Correction to ‘MRE11 UFMylation promotes ATM activation’

**DOI:** 10.1093/nar/gkae802

**Published:** 2024-09-12

This is a correction to: Zhifeng Wang, Yamin Gong, Bin Peng, Ruifeng Shi, Dan Fan, Hongchang Zhao, Min Zhu, Haoxing Zhang, Zhenkun Lou, Jianwei Zhou, Wei-Guo Zhu, Yu-Sheng Cong, Xingzhi Xu, MRE11 UFMylation promotes ATM activation, *Nucleic Acids Research* 2019, 47(8):4124-4135, https://doi.org/10.1093/nar/gkz110

The authors have found that an error was introduced in the INPUT panel in Figure 2B during figure assembly. The Tubulin blot was erroneously duplicated from NBS1. The band of Tubulin has been substituted for the correct one in the new ‘INPUT’ panel of Figure 2B provided below.

The original data is supplied as supplementary material.

This correction does not affect the results, discussion and conclusions presented in the article. These details have been corrected only in this correction notice to preserve the published version of record.

The new ‘INPUT’ panel of Figure 2B:


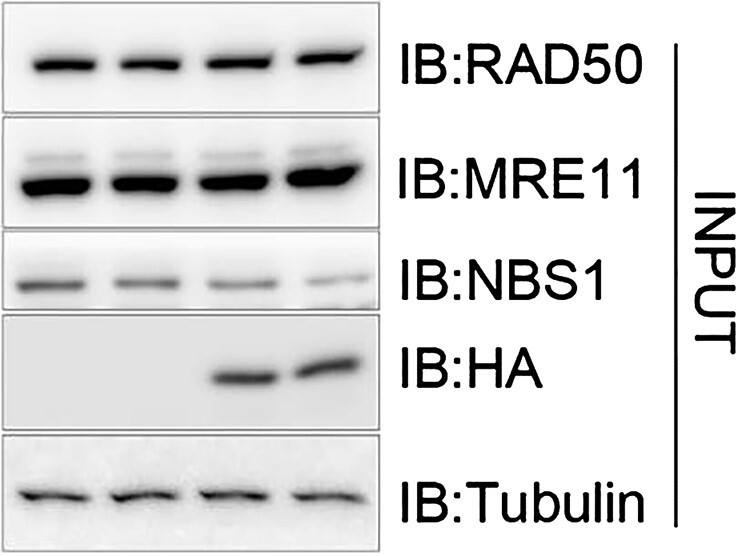



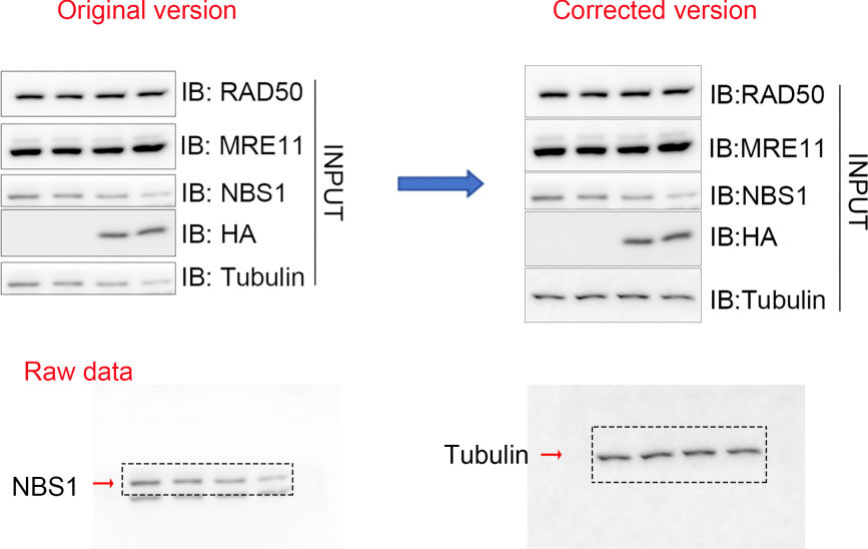


## Supplementary data

Supplementary Data are available at NAR Online.

## Supplementary Material

gkae802_Supplemental_Files

