# Supplementary material for: MRE11 UFMylation promotes ATM activation
Source: Nucleic Acids Res. 2019 Feb 20;47(8):4124–35. doi: 10.1093/nar/gkz110 (PMC6486557; doi:10.1093/nar/gkz110)

Supplementary Material

Supplementary figure legends

Supplementary Figure 1 UFMylation is involved in the DDR

A. UFL1 was recruited to DNA damage stripes. U2OS cells were micro-irradiated with a UV laser (365 nm), then fixed with 4% PFA for 5 min at room temperature followed by ice-cold methanol at 4°C for 20 min, and then co-immunostained with antibodies against UFL1 (green) and γH2AX (red). **B. DNA damage induced an increase of UFL1 loading onto chromatin.** U2OS cells were treated with 10 μg/ml bleomycin or exposed to 5 Gy X-ray irradiation. Chromatin fractionation assays were performed at the indicated times.

Supplementary Figure 2 UFL1/UfSP2 dynamically interacts with the MRN complex in response to ionizing radiation (IR)

A. IR-induced interaction between HA-UFL1 and GFP-MRE11. HEK293T cells co-expressing HA-UFL1 and GFP-MRE11 were irradiated at a dose of 5 Gy. Total cell lysates were harvested at the indicated time points after IR and subjected to immunoprecipitation and immunoblotting with the indicated antibodies. **B. IR disrupted the interaction between HA-UfSP2 and GFP-MRE11.** HEK293T cells co-expressing HA-UfSP2 and GFP-MRE11 were irradiated at a dose of 5 Gy. Total cell lysates were harvested at different times as indicated, and subjected to immunoprecipitation and immunoblotting with the indicated antibodies.

Supplementary Figure 3 UFMylation promotes MRE11 recruitment to the DNA damage stripes

A & B. UFL1 depletion decreased the initial recruitment of NBS1 to DNA damage

stripes. UFL1-depleted (siUFL1) or mock (siCTR)-depleted DU145 cells transiently expressed NBS1-GFP. **C & D. Over-expression of UfSP2 decreased the initial recruitment of NBS1 to DNA damage stripes.** DU145 cells were co-transfected with a HA vector, HA-UfSP2 or HA-UfSP2(C302S) and NBS1-GFP at a molar ratio of 10:1. GFP-positive cells were micro-irradiated with a UV laser (365 nm) and consecutive images were captured at 10-sec intervals for 10 min. Representative images of GFP-MRE11 recruitment are shown in A and C, and statistical analyses of the recruitment dynamics with the Spearman's rank-order correlation test are shown in B and D.

Supplementary Figure 4 Recruitment dynamics of UFL1 to the DNA damage site. U2OS cells were exposed to 5-Gy X-ray irradiation, fixed at the indicated timepoints after IR, and subjected to immunofluorescence staining with the indicated antibodies. A. Representative images of UFL1 foci and γH2AX foci are shown. B. Representative images of MRE11 foci and γH2AX foci are shown. C. The number of UFL1 foci or MRE11 foci per cell that colocalized with γH2AX foci from at least 30 individual cells was determined and statistically analyzed by two-way ANOVA.

Supplementary Figure 5 MRE11 is required for optimal recruitment of UFL1 to the DNA damage site. A549 cells stably expressing shCTR or shMRE11 were re-introduced with FLAG-MRE11 or FLAG-MRE11(K282R) or FLAG-MRE11(G285C). The cells were treated with 5-Gy IR, then fixed after 10 min, and subjected to immunofluorescence staining with the indicated antibodies. A. Representative images of UFL1 foci and γH2AX foci are shown. B. The number of UFL1 foci per cell that colocalized with γH2AX foci from at least 30 individual cells was determined and statistically analyzed by a two-way ANOVA test. MRE11 knockdown efficiency is also

shown.

Supplementary Figure 6 UFMylation-defective mutants compromised DNA damage-induced ATM activation. A549 cells stably expressing shCTR or shMRE11 were re-introduced with FLAG-MRE11 or FLAG-MRE11(K282R) or FLAG-MRE11(G285C). The cells were treated with 5-Gy IR. Total cell lysates were harvested 20 min after IR and analyzed by SDS-PAGE and immunoblotting with the indicated antibodies.

Wang et al., Supplementary Figure 1

A

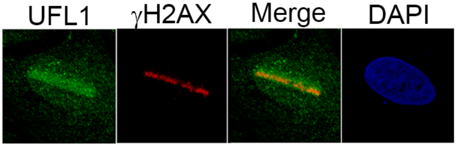

B

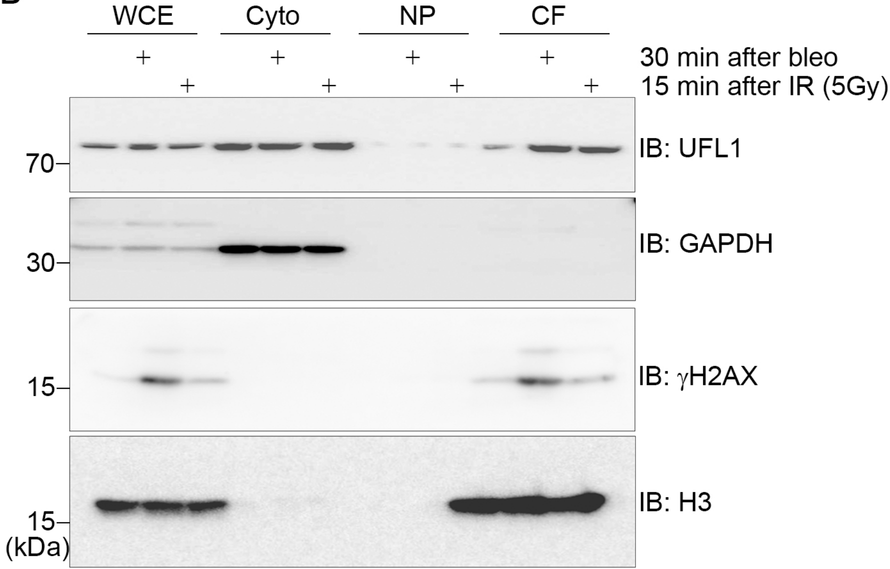

Wang et al., Supplementary Figure 2

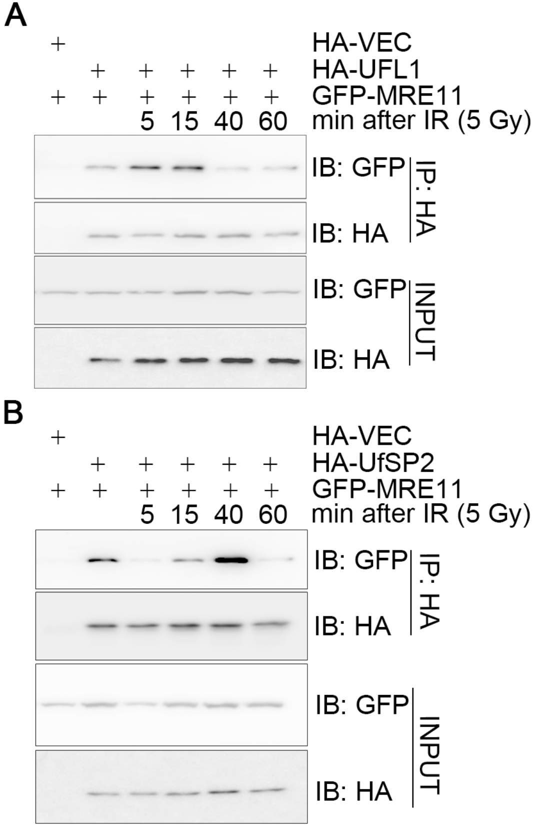

Wang et al., Supplementary Figure 3

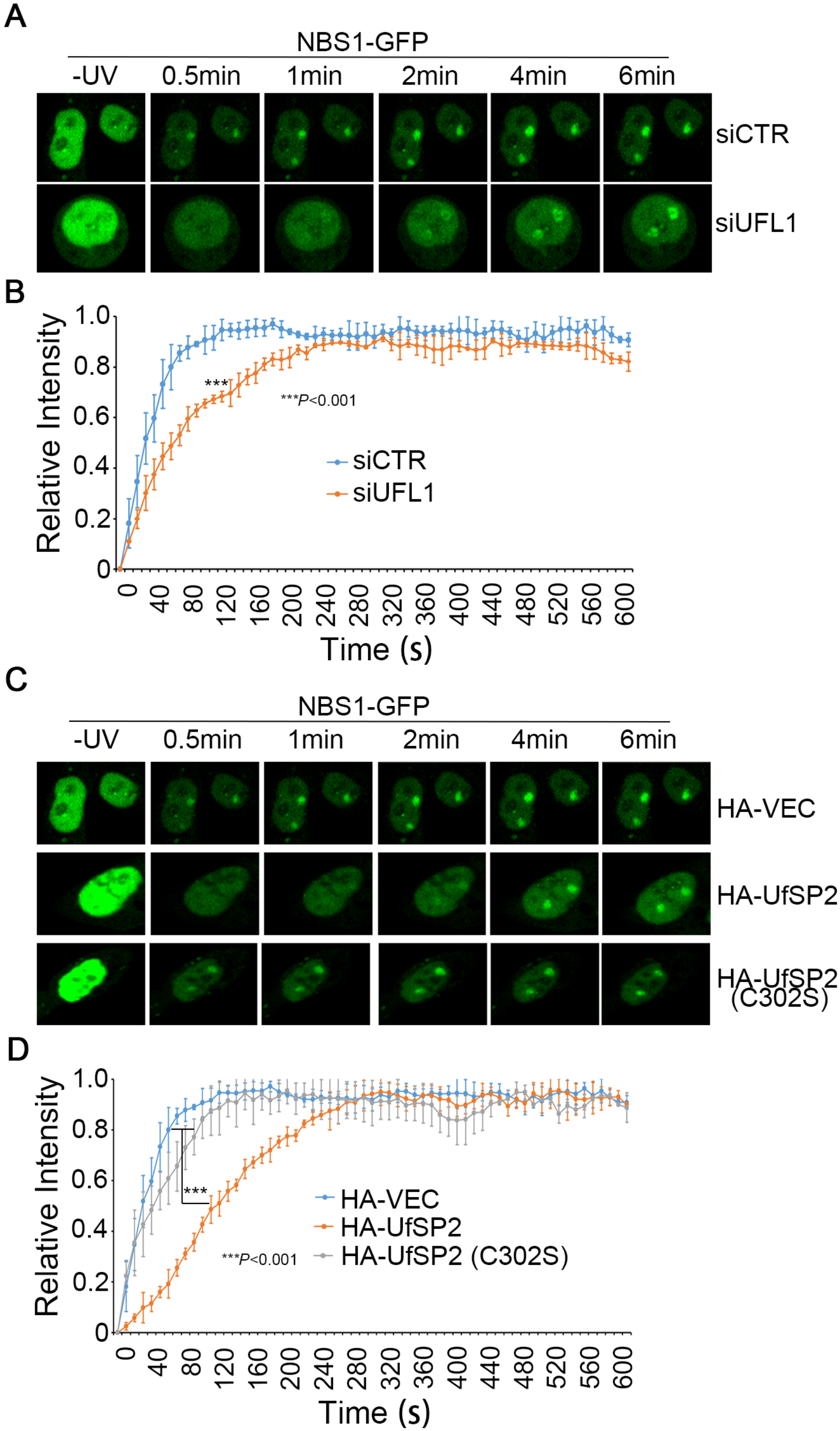

Wang et al., Supplementary Figure 4

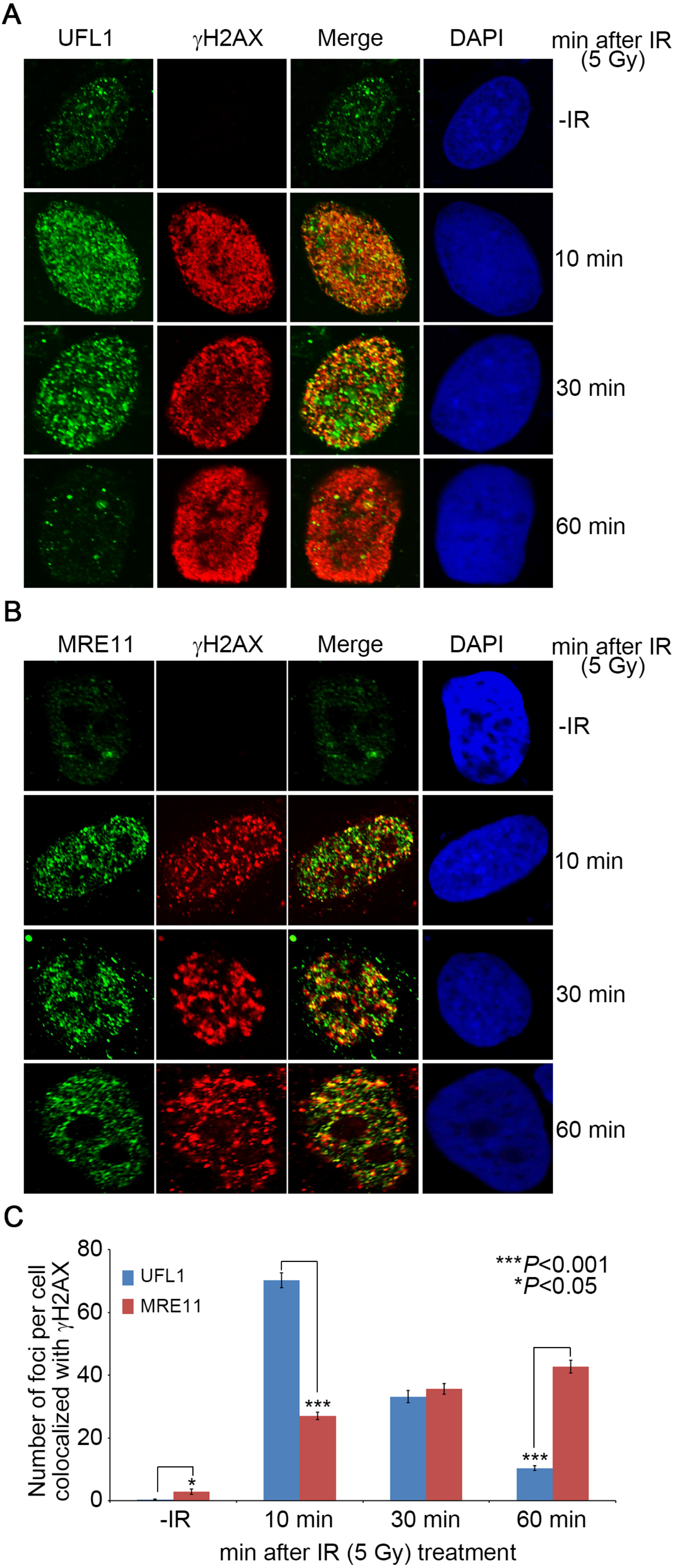

Wang et al., Supplementary Figure 5

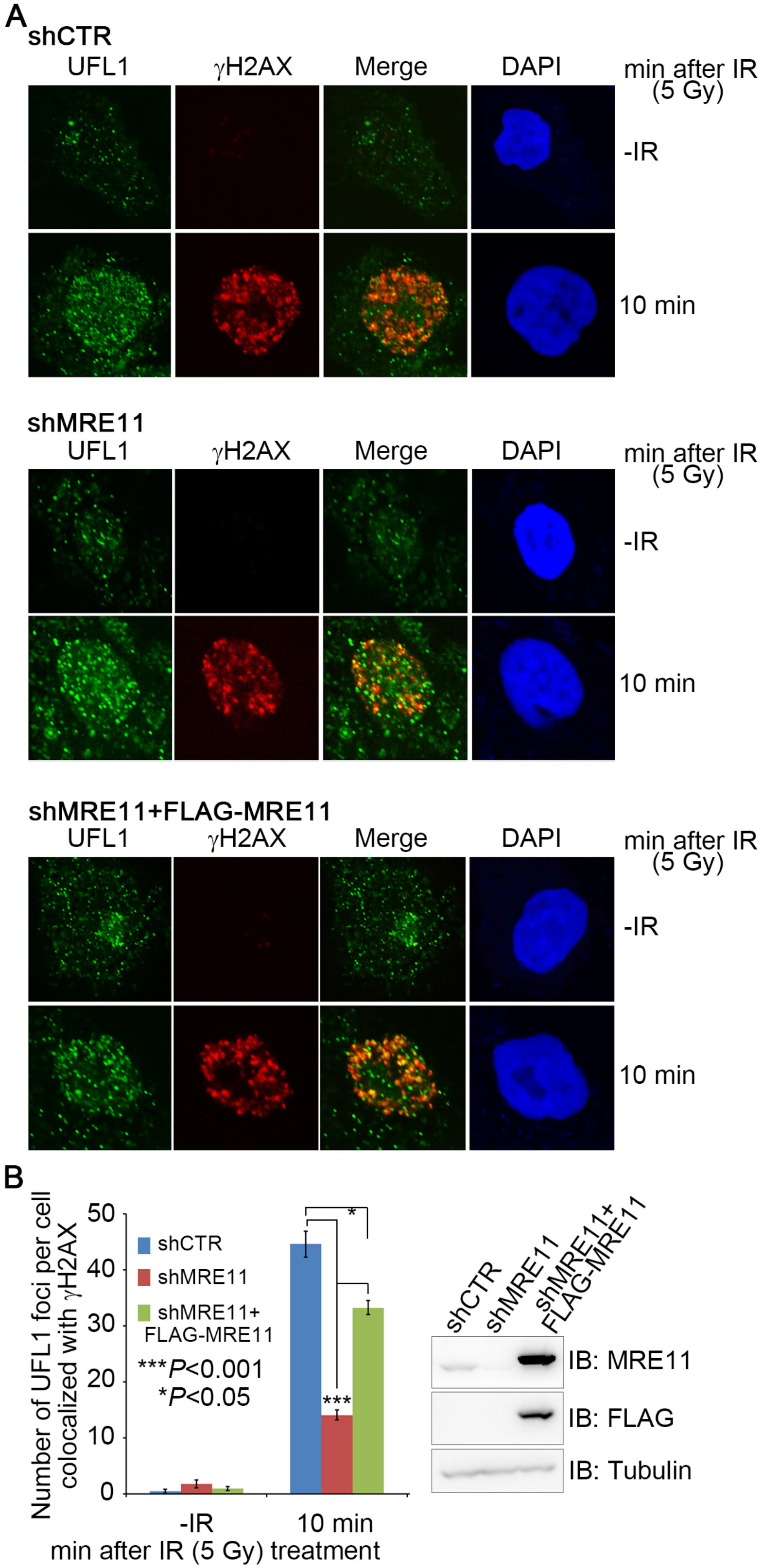

Wang et al., Supplementary Figure 6

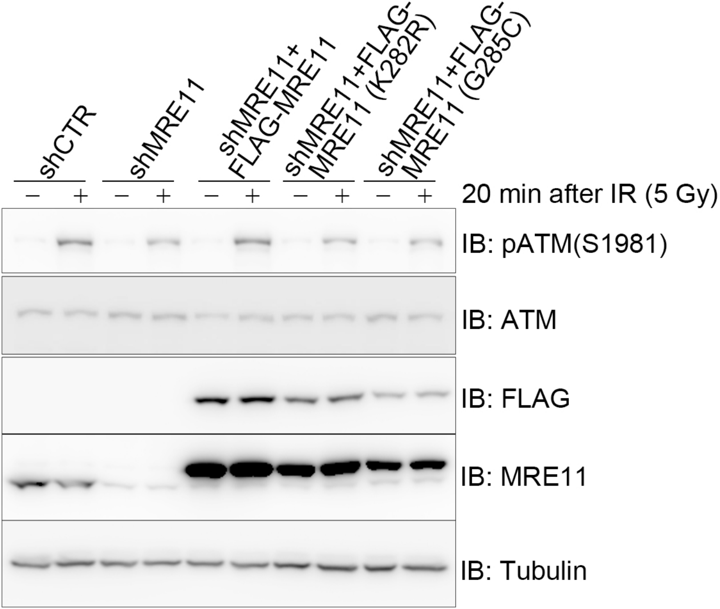

Supplement: Supplementary Data [file gkz110_supplemental_file.pdf]
