# Supplementary figures and images for: Correction to ‘MRE11 UFMylation promotes ATM activation’
Source: Nucleic Acids Res. 2024 Sep 12;52(18):11412. doi: 10.1093/nar/gkae802 (PMC11472032; doi:10.1093/nar/gkae802)

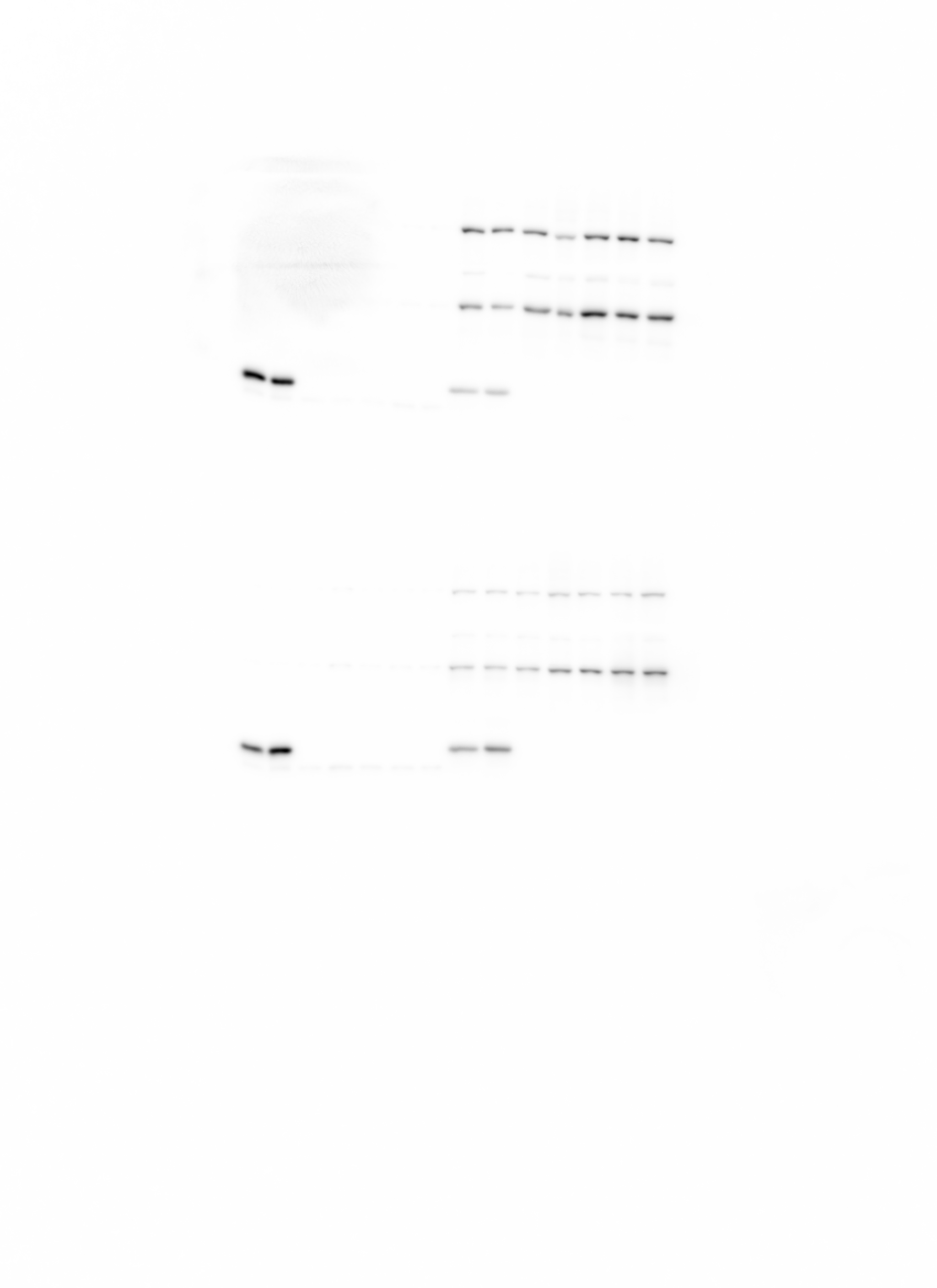

Supplement: gkae802_Supplemental_Files [file gkae802_supplemental_files.zip › HA.tif]

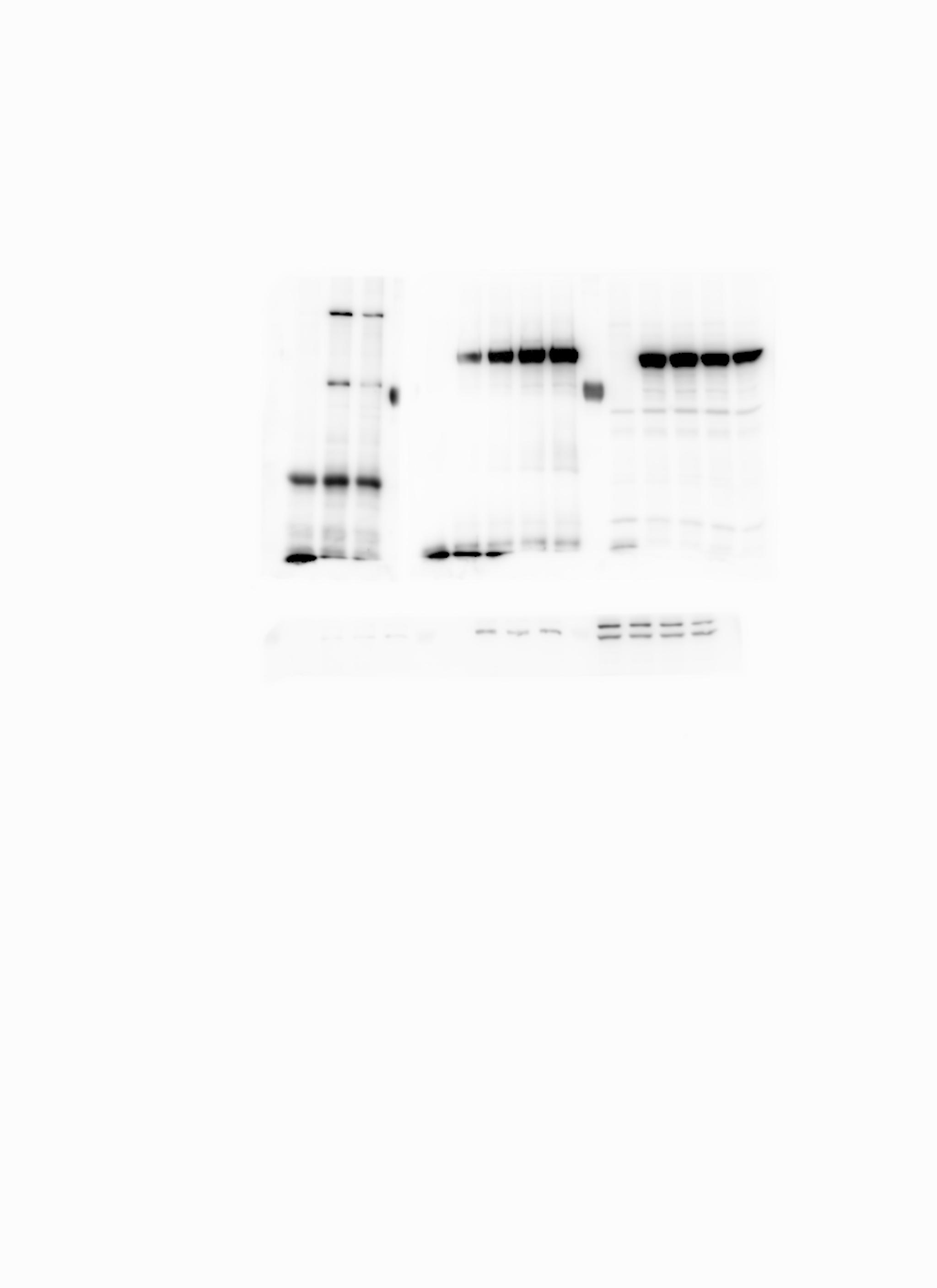

Supplement: gkae802_Supplemental_Files [file gkae802_supplemental_files.zip › NBS1.jpg]

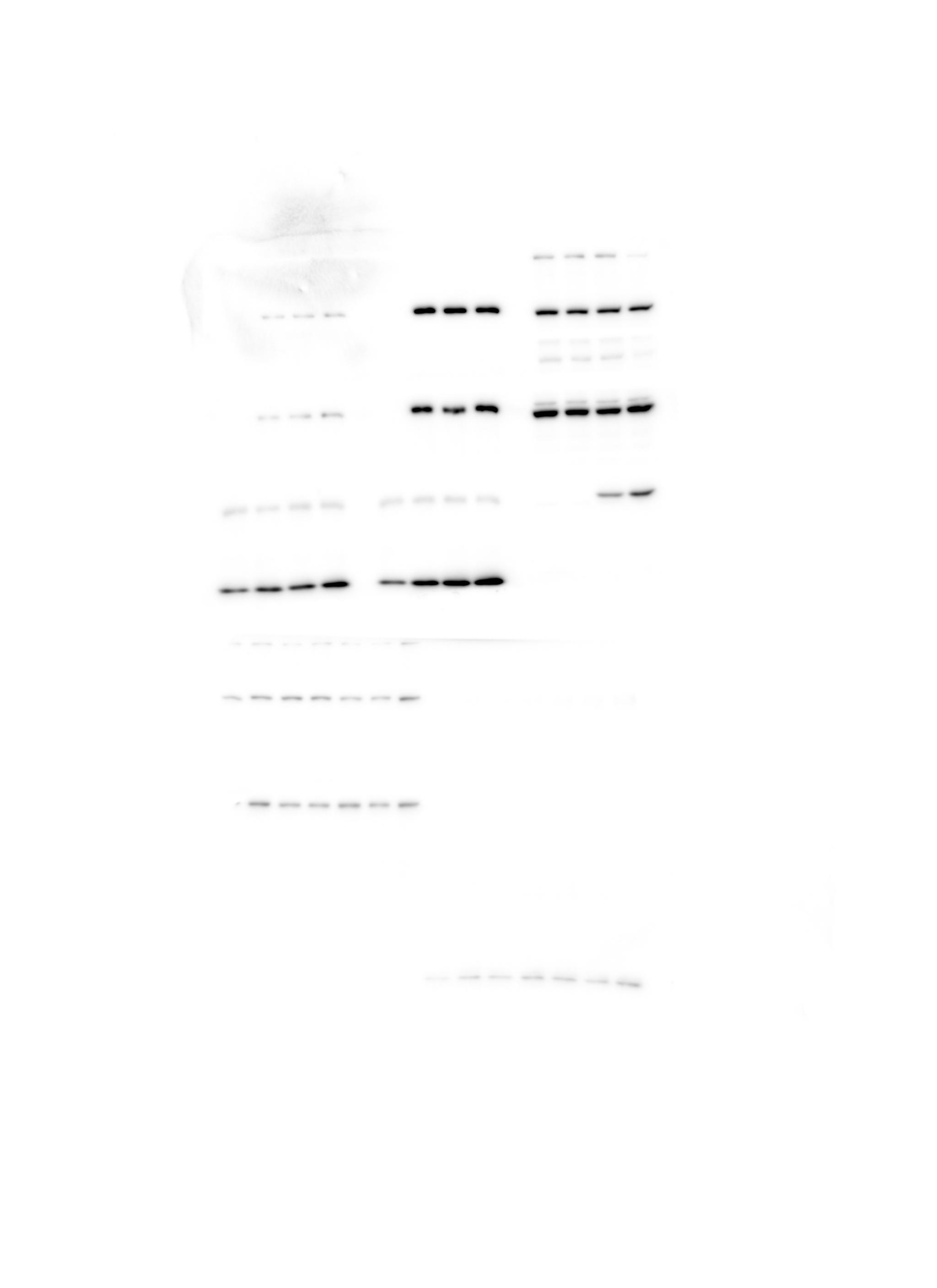

Supplement: gkae802_Supplemental_Files [file gkae802_supplemental_files.zip › RAD50 and MRE11.jpg]

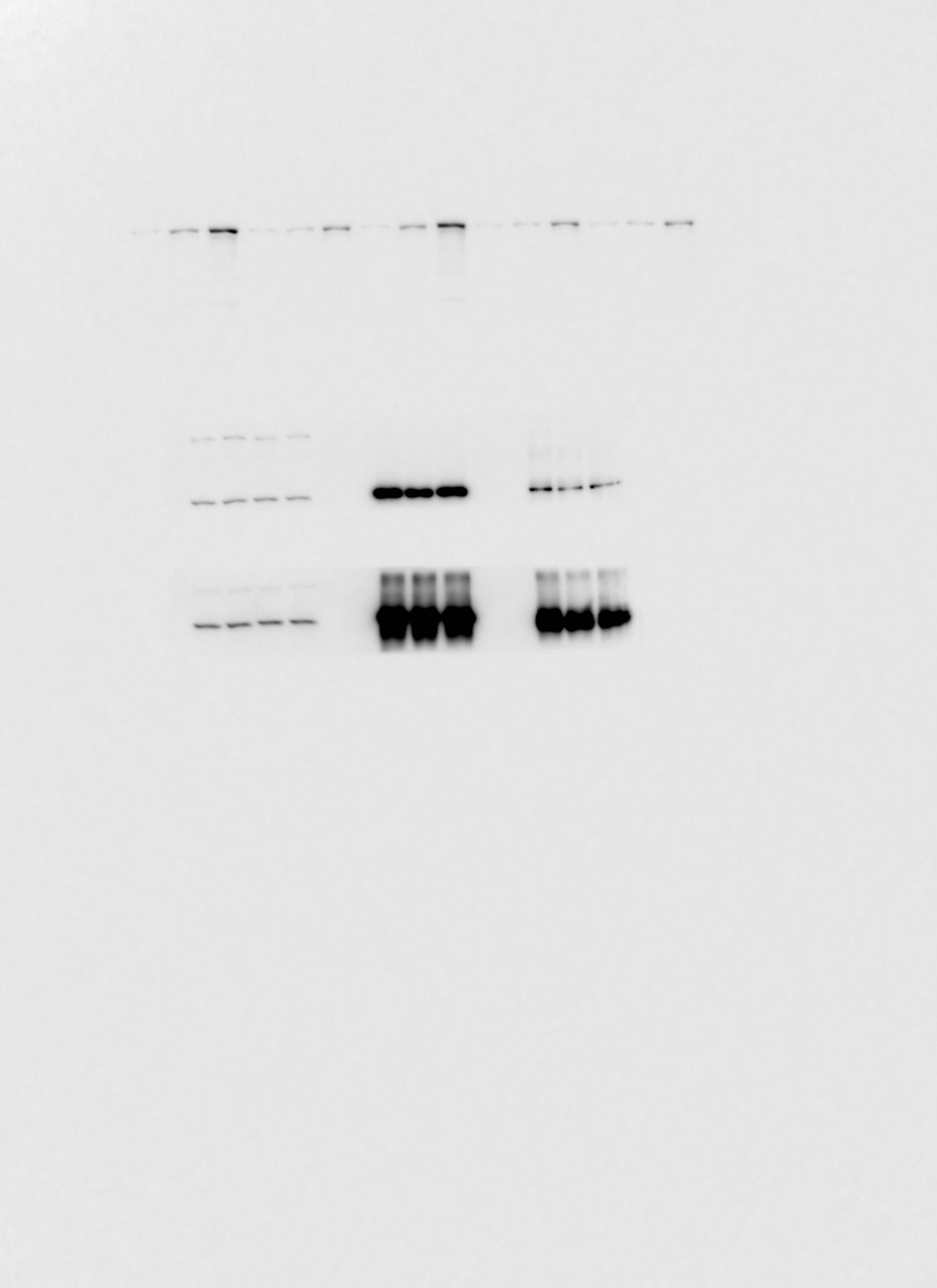

Supplement: gkae802_Supplemental_Files [file gkae802_supplemental_files.zip › Tubulin.jpg]
